# Diabetes negatively affects cortical and striatal GABAergic neurons: an effect that is partially counteracted by exendin-4

**DOI:** 10.1042/BSR20160437

**Published:** 2016-12-05

**Authors:** Martin Larsson, Grazyna Lietzau, David Nathanson, Claes-Göran Östenson, Carina Mallard, Maria E. Johansson, Thomas Nyström, Cesare Patrone, Vladimer Darsalia

**Affiliations:** * Department of Clinical Science and Education, Södersjukhuset, Internal Medicine, Karolinska Institutet, 118 83 Stockholm, Sweden; † Department of Anatomy and Neurobiology, Medical University of Gdansk, 80 221 Gdansk, Poland; ‡ Department of Molecular Medicine and Surgery, Karolinska Institutet, 171 76 Stockholm, Sweden; § Department of Physiology, Institute of Neuroscience and Physiology, The Sahlgrenska Academy, University of Gothenburg, 405 30 Gothenburg, Sweden

**Keywords:** γ-aminobutyric acid (GABA), diabetes, exendin-4, glucagon-like peptide-1 receptor (GLP-1R), interneurons, neurological complications

## Abstract

Type 2 diabetic (T2D) patients often develop early cognitive and sensorimotor impairments. The pathophysiological mechanisms behind these problems are largely unknown. Recent studies demonstrate that dysfunctional γ-aminobutyric acid (GABAergic) neurons are involved in age-related cognitive decline. We hypothesized that similar, but earlier dysfunction is taking place under T2D in the neocortex and striatum (two brain areas important for cognition and sensorimotor functions). We also hypothesized that the T2D-induced effects are pharmacologically reversible by anti-diabetic drugs targeting the glucagon-like peptide-1 receptor (GLP-1R). We determined the effect of T2D on cortical and striatal GABAergic neurons positive for glutamic acid decarboxylase-67 (GAD67), calbindin (CB), parvalbumin (PV) and calretinin (CR) by using immunohistochemistry and quantitative microscopy. Young and middle-aged T2D Goto-Kakizaki (GK) (a model of spontaneous T2D) and Wistar rats were used. Furthermore, we determined the therapeutic potential of the GLP1-R agonist exendin-4 (Ex-4) by treating middle-aged GK rats for 6 weeks with 0.1 μg/kg Ex-4 twice daily. We show that T2D reduced the density of GAD67-positive neurons in the striatum and of CB-positive neurons in both striatum and neocortex. T2D also increased the average volume of PV-positive interneurons in the striatum. Ex-4 treatment increased the density of CB-positive neurons in the striatum of GK rats. Our data demonstrate that T2D negatively affects GAD67 and CB-positive GABAergic neurons in the brain during aging, potentially identifying some of the pathophysiological mechanisms to explain the increased prevalence of neurological complications in T2D. We also show a specific, positive effect of Ex-4 on striatal CB-positive neurons, which could be exploited in therapeutic perspective.

## Abstract (precis)

Diabetes negatively affects specific subtypes of inhibitory neurons in brain areas that regulate sensory and motor functions. This impairment can be partially reversed by exendin-4 (Ex-4). The findings could contribute to the development of treatments against diabetic neurological complications.

## INTRODUCTION

Over 350 million adults worldwide were living with Type 2 diabetes (T2D) in 2015 [1,2]. Adverse changes in the metabolism associated with T2D can be harmful to many organ systems including the nervous system [3,4]. However, although the peripheral nervous system complications of T2D have been extensively studied and characterized [5], less is known about the functional and anatomical effects of T2D on the central nervous system (CNS).

The most common CNS disorder associated with T2D is stroke. The risk of stroke is doubled in T2D [6,7] with more severe neurological impairments and a lesser degree of recovery than in non-diabetic patients [8]. The exact causes of decreased neurological recovery in T2D after stroke are unknown, but could be linked to pre-existing pathological alterations in the brain at cellular and structural levels. This hypothesis is also reinforced by the observation that the likelihood of early development of age-associated neurological complications, such as different forms of cognitive impairment and dementias [including Alzheimer's diseases (AD)] is dramatically increased in T2D [9–14]. Imaging studies have also confirmed the negative impact of T2D on the brain at structural level as shown by detectable cerebral atrophy in T2D patients [15,16].

Despite the strong association between T2D and CNS complications, the specific brain structures or neuronal cell types that are affected by T2D have not yet been precisely identified. Furthermore, the majority of preclinical research in the field has mainly focused on the hippocampus and studied the co-morbid effects of T2D in animal models of neurodegenerative disorders such as AD [13,17–19]. However, previous clinical data show a broad range of additional cognitive and sensorimotor impairments in T2D patients without AD [9]. Furthermore, Parkinson's disease (PD) patients show faster development and more severe motor dysfunction in presence of T2D [20]. Thus, brain areas other than hippocampus also need to be thoroughly investigated.

Recent studies of age-related cognitive decline demonstrate the involvement of dysfunctional γ-aminobutyric acid (GABAergic) interneurons [21] and their increased susceptibility under metabolic stress [22]. Moreover, studies have reported selective changes in subtypes of GABAergic interneurons in the hippocampus [23] and piriform cortex of diabetic rats [24], two brain areas involved in memory and olfaction respectively. Whether similar and/or additional alterations in GABAergic neurons are present in other brain areas is unknown. To this end, it is particularly interesting whether cognitive and sensorimotor impairments in T2D could be related to pathological alterations in neocortical and striatal neuronal circuits since these brain areas regulate these functions. Approximately 5–10% of the neuronal population in neocortex and striatum is constituted of GABAergic interneurons, which exert significant modulatory effects on the normal functioning of these structures [25,26]. A subgroup of interneurons is characterized by the expression of the calcium-binding proteins (CaBPs) calbindin (CB)-D 28kD, calretinin (CR) and parvalbumin (PV) [27]. In a study by Castillo-Gomez et al. [28] using the streptozotocin-induced Type 1 diabetic model, the authors have shown that in the medial prefrontal cortex diabetes reduced the levels of glutamic acid decarboxylase-67 (GAD67), which is the principal enzyme responsible for GABA synthesis and that this reduced expression correlated to depressive-like behaviour [28]. Whether similar alterations are induced by T2D and whether they could be linked to impairment of neocortical and striatal function is unknown.

From a therapeutic perspective, no option is currently available to treat CNS neuropathology in T2D. We have recently shown that T2D decreases the number of CB-positive interneurons in the piriform cortex of the T2D rat and that this effect can be counteracted by the treatment with the glucagon-like peptide-1 receptor (GLP-1R) agonist exendin-4 (Ex-4) [24]. Ex-4 is a stable synthetic form of GLP-1R that induces glucose-dependent insulin secretion and inhibits glucagon release in the pancreas [29]. For these properties, it has been developed for clinical treatment of T2D [29,30]. Besides its anti-diabetic properties, Ex-4 can cross the blood brain barrier [31] and preclinical works have demonstrated neuroprotective efficacy of Ex-4 and other GLP-1R analogues in several neurological disorders (reviewed in [32–34]). Whether such treatment could prove beneficial against potential interneuron pathology in the neocortex and the striatum in T2D has not yet been investigated. Interestingly, a recent work by Korol et al. [35] showed that GLP-1R activation enhances GABA-signalling in the hippocampus by pre- and postsynaptic mechanisms.

The goal of our study was to determine whether T2D affects neocortical and striatal GABAergic neurons during aging and to evaluate the therapeutic potential of GLP-1R activation in reversing the identified alterations. As a model of T2D, we used the Goto-Kakizaki (GK) rat, which is a non-obese rat model of T2D derived from the Wistar strain that spontaneously develops T2D [36] accompanied by common T2D complications often observed in human patients [37,38].

## MATERIALS AND METHODS

### Animals and experimental groups

GK rats were used as an experimental model of T2D (see above). Non-diabetic age-matched Wistar rats were used as controls. The rats were housed in 12/12h light/dark cycle and were given free access to food and water. All experiments were conducted in accordance with the “Guide for the Care and Use of Laboratory Animals” published by U.S. National Institutes of Health and approved by the local ethics committee.

**Study 1**. To evaluate the effect of T2D on CNS GABAergic neurons during aging, 13-month-old T2D GK (*n*=6) and non-diabetic Wistar (*n*=6) rats were used. Young adult (3-month-old) GK (*n*=7) and Wistar (*n*=6) rats were used as controls.

**Study 2.** To determine the therapeutic potential of Ex-4 in reversing T2D-induced neuropathological changes, we used 9-month-old GK rats. GK rats were treated with 0.1 μg/kg Ex-4 intraperitoneally (i.p.) twice daily for 6 weeks (*n*=8) or vehicle (*n*=10), before killing. Dose and dosing regimen were chosen to mimic clinical application of Ex-4 treatment.

### Monitoring of T2D and treatment effects on glycaemia

In Study 1, GK and Wistar rats at 3 and 13 months of age had monitored for fasted (6 h) blood glucose and plasma insulin levels. Three-month-old GK rats showed slightly, but significantly higher fasting glycaemia as compared with Wistar rats (approximately 9mM compared with 6mM), whereas 13-month-old GK rats showed very high levels of hyperglycaemia (approximately 18mM). Plasma insulin levels were significantly lower in GK rats already at 3 months as compared with age-matched Wistar controls (approximately 2μg/l compared with 4μg/l). At 13 months, the insulin levels decreased even further in GK rats (less than 1μg/l). The glycaemic data of Study 1 are presented in our recent publication [39]. In Study 2, 9-month-old GK rats were treated with Ex-4 or vehicle for 6 weeks before killing. Ex-4 significantly decreased blood glucose (approximately 6mM compared with 10mM), and increased insulin secretion (approximately 2μg/l compared with 1.5 μg/l). The glycaemic data of Study 2 have been recently published [24].

Blood glucose levels were measured in all animals using a glucometer after 6 h fasting with free access to water. Insulin was measured by a rat insulin ELISA kit (kindly provided by Crystal Chem).

### Immunohistochemistry

Animals were deeply anesthetized with sodium pentobarbital and transcardially perfused with 4% paraformaldehyde (PFA). The brains were extracted and after overnight post-fixation in 4% PFA put in 25% sucrose in phosphate buffer until they sank. Brains were cut in 40 μm thick coronal and sagittal sections using one hemisphere for each plane of sectioning. Nissl substance was stained by using 0.1% Cresyl Violet acetate (Sigma–Aldrich). For immunohistochemical staining, the following primary antibodies were used: rabbit anti-Parvalbumin (1:1500, Abcam), rabbit anti-Calbindin- D28k (1:1500, Abcam), rabbit anti-Calretinin (1:1500, Vector Laboratories) and mouse anti-GAD-67 (1:500, Merck Millipore). Antigen retrieval was performed using citrate buffer or EDTA. Primary antibodies were visualized using biotin-conjugated secondary antibodies (1:200, Vector Laboratories) after peroxidase substrate reaction (ABC kit, Vector Laboratories) as previously described [39]. The same makers were measured in both Study 1 and 2.

### Quantitative analysis

**Figure 1:**
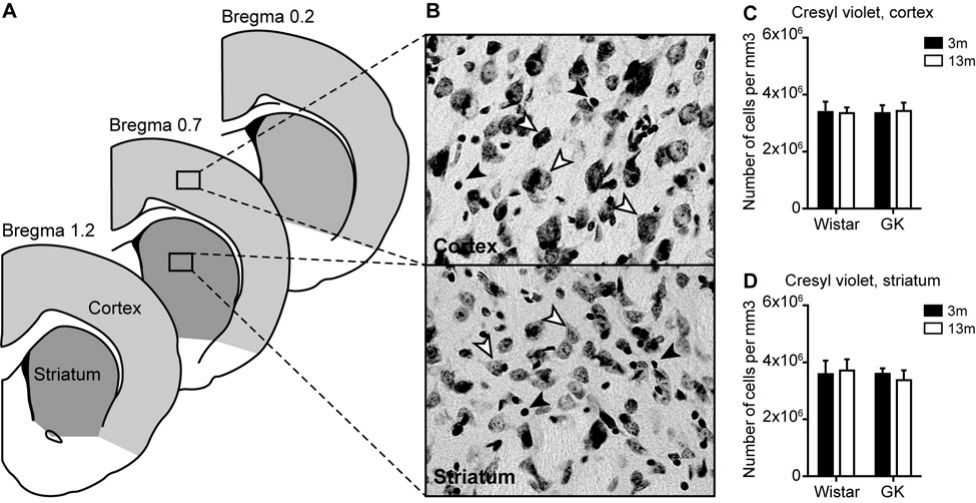
The effects of T2D on the general neuronal density in the neocortex and the striatum during aging (**A**) Representation of the brain sections where the cell quantifications were performed. (**B**) The representative images of Nissl staining in the cortex and striatum. White arrowhead indicates an example of a cell with neuronal morphology. Black arrowhead indicates an example of a cell with glial morphology. The density of neurons in the neocortex (**C**) and the striatum (**D**) respectively. Two-way ANOVA was used to determine whether diabetes modified the effect of normal aging observed in Wistar rats. Two-way ANOVA was followed by Tukey's multiple comparison test to determine the differences between experimental groups; 3 months old (3m), 13 months old (13m).

Cells were counted using a computerized setup (NewCast softwareVisiopharm), connected to Olympus BX51 epifluorescent/light microscope (Olympus). The number of Nissl, CB, CR, PV and GAD67-positive cells were counted on three evenly spaced (distance 0.5 mm) coronal sections in each animal starting at 1.2 mm anterior to Bregma (Figure 1A). Separate counts were made in both the striatum and the cortex. Cortex and striatum were delineated using the computer-assisted stereology toolbox on the three sections. Counting was carried out using a counting frame that moved at evenly spaced intervals (steps) from a random starting point (determined by the NewCast software) over the total delineated area. Counting was perfumed by the investigator blinded to experimental groups. The step length was chosen so that approximately 100–200 cells in each animal were counted. The total cell number in the three sections was estimated using the following formula: Total cell number=(Counted number × Step area)/Counting frame area. From the estimated total cell number, the cell density within the sampled brain volume was determined. The data are presented as the number of cells per mm^3^.

The counting of GAD67+ cells, in Study 1, was performed using sagittal sections due to limited tissue availability; three evenly spaced sections (distance 0.5 mm) starting at 2.0 mm lateral from midline were used. For the cortical GAD67 counts in the sagittal sections, the areas between 1.2 mm and 0.2 mm anterior from Bregma were used in order to match coronal planes used for other assessments.

Cell volume estimates were made using the nucleator technique [40].

### Cytokine assay

Serum cytokine levels were measured in rats treated with or without Ex-4 for 6 weeks. Levels of IL-1β, MCP-1, IL-6, IL-10 and TNFα were simultaneously measured using the Bio-Plex Multiplex Cytokine Assay (Bio–Rad Laboratories) according to the manufacturer's protocol. Samples below detection limit were assigned a value corresponding to half of the sensitivity of the assay (Assay sensitivity: IL-1β, 2pg/ml; MCP-1, 3pg/ml; IL-6, 10 pg/ml and TNFα, 3 pg/ml).

### Statistics

Homoscedasticity (homogeneity of variance) was tested by using Breusch–Pagan test and data plotting. Shapiro–Wilk test and Q–Q plot were used to test for normal distribution of residuals. Presence of outliers was analysed using Q–Q plot and Cook's distance. In the total data set, three outliers were detected in different animals and different markers. Final statistical analysis was made using GraphPad Prism 6.

In Study 1, two-way ANOVA was used to determine whether diabetes modified the effect of normal aging observed in Wistar rats. Two-way ANOVA was followed by Tukey's multiple comparisons test to determine the differences between experimental groups. In the figures, the asterisks indicate significant differences between age groups within each strain and the hash symbol shows where the effects of aging were modified by diabetes.

In Study 2, Student's *t* test was used. Data are expressed as mean +/− S.D. *P*-value less than 0.05 was considered significant in both studies.

## RESULTS

### T2D does not affect the total density of neurons but reduces the density of GAD67-positive cells in the striatum

To quantify the density of neurons in the cortex and the striatum, the sections were stained for Nissl substance by Cresyl Violet. Since Cresyl Violet acetate also stains myelin cells, for neuron estimation only the cells with clear neuronal morphology were counted (Figure 1B). There was no change with age in the total neuronal density in either GK or Wistar rats in neocortex and striatum (Figures 1C and 1D).

**Figure 2:**
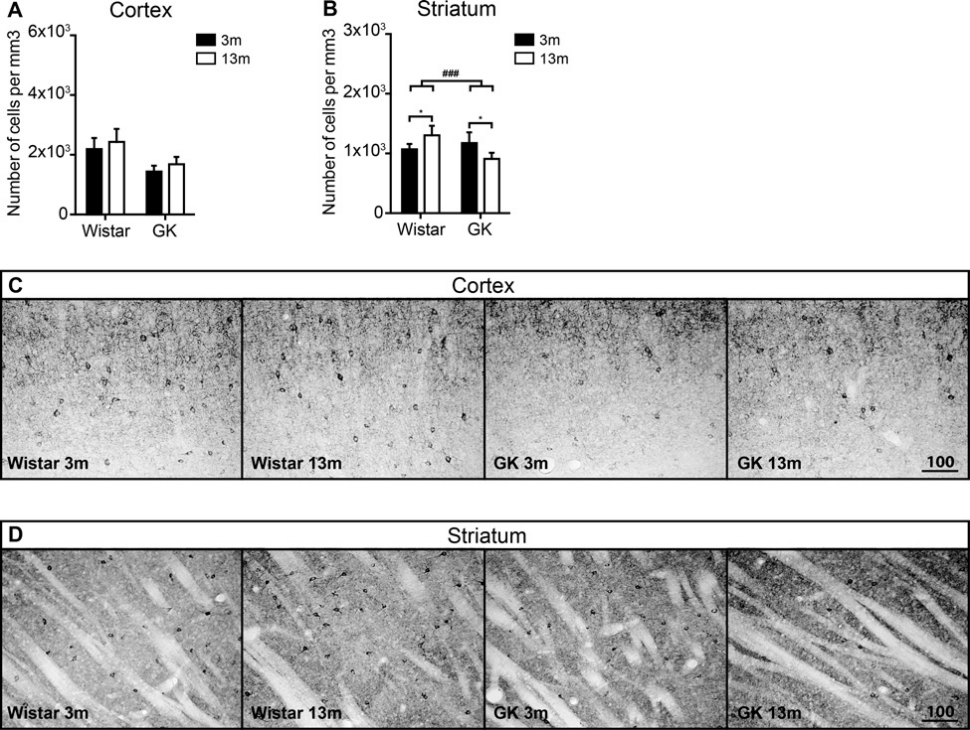
The effects of T2D on the density of GAD67+ neurons in the neocortex and the striatum during aging The density of GAD67+ cells the neocortex (**A**) and the striatum (**B**). Representative images of GAD67 immunoreactivity in the neocortex (**C**) and the striatum (**D**). Scale bar on panels (C) and (D) equals 100 μm. Two-way ANOVA was used to determine whether diabetes modified the effect of normal aging observed in Wistar rats. The hash (#) symbol shows where the effects of aging were modified by diabetes. ### denotes *P*<0.001. Two-way ANOVA was followed by Tukey's multiple comparison test to determine the differences between experimental groups. Error bars indicate means±S.D.; * denotes *P*<0.05; 3 months old (3m), 13 months old (13m).

In the neocortex, GAD67-positive cells were counted in all layers except layer 4 where intense background staining did not allow for accurate quantification. The results of the two-way ANOVA showed no statistically significant interaction between age and diabetes. However, T2D GK rats showed lower density of GAD67-positive cells in the neocortex in comparison with Wistar rats, both at 3 and at 13 months (Figures 2A and 2C) indicating a strain difference.

The result of the density measurements of GAD67-positive cells in the striatum was different compared with the neocortex. There was a significant interaction between age and diabetes in the striatum by two-way ANOVA analysis (*P*<0.001). In Wistar rats, the density of GAD67-positive neurons was significantly increased during aging (*P*<0.05) (Figures 2B and 2D). The opposite was observed in T2D GK rats, where the density of GAD67-positive neurons was significantly reduced during aging (*P*<0.05) (Figures 2B and 2D). These results indicate that the decreased density of GAD67-positive cells in aged GK rats was a diabetic and not an aging effect.

### T2D reduces the density of CB-positive neurons in the neocortex and striatum

**Figure 3:**
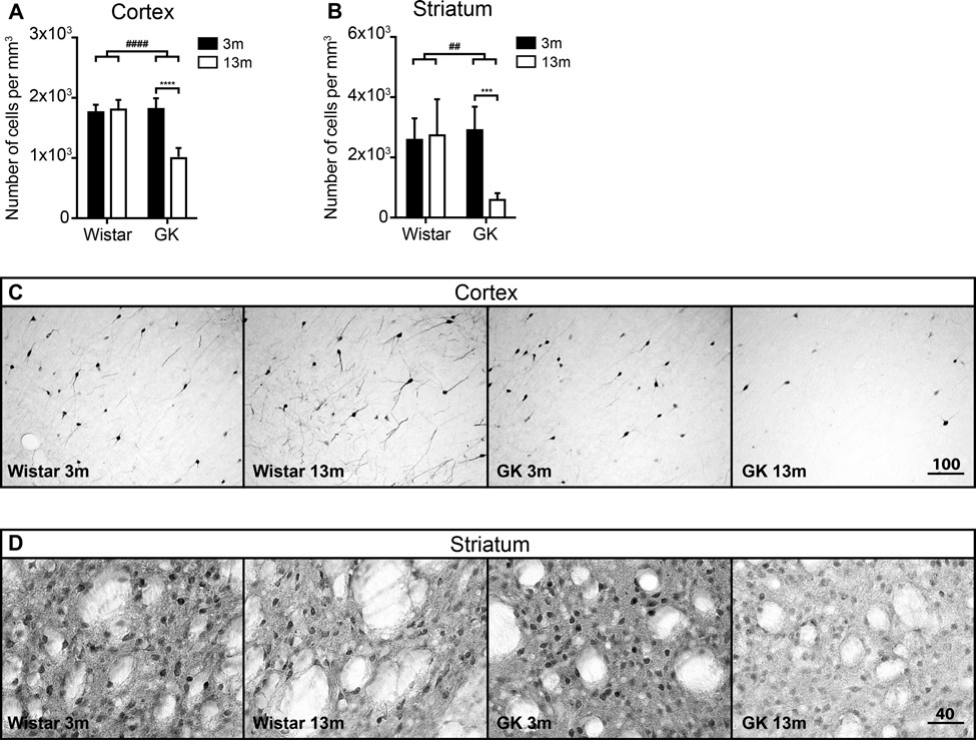
The effects of T2D on the density of CB-positive cells in the neocortex and the striatum during aging The density of CB-positive neurons in the neocortex (**A**) and the striatum (**B**). The representative images of CB immunoreactivity in the neocortex (**C**) and the striatum (**D**). Scale bar on panels (C) and (D) equals 100 μm and 40 μm respectively. Two-way ANOVA was used to determine whether diabetes modified the effect of normal aging observed in Wistar rats. The hash (#) symbol shows where the effects of aging were modified by diabetes. ## denotes *P*<0.01, ####*P*<0.0001. Two-way ANOVA was followed by Tukey's multiple comparison test to determine the differences between experimental groups. Error bars indicate means±S.D.; *** denotes *P*<0.001, ****P<0.0001; 3 months old (3m), 13 months old (13m).

The assessment of the density of CB-positive neurons showed a highly significant interaction between age and diabetes in the two-way ANOVA analysis (*P*<0.0001) indicating the effect of diabetes on this marker. The 13-month-old GK rats showed a significant decrease in CB-positive cell density in the neocortex (approximately 45% less) compared with 3-month-old GK rats (*P*<0.0001), with a visible decrease in the number of neurites as well (Figures 3A and 3C). On the contrary, the Wistar rats showed no difference with age in the density of CB-positive cells (Figures 2A and 2C).

Similarly to neocortex, the test for interaction between age and diabetes in the striatum showed a significant effect of diabetes (*P*<0.01). The density of CB-positive neurons was reduced in 13-month-old GK rats to approximately 20% of that of 3-month-old GK rats (*P* ≤ 0.001) (Figures 3B and 3D). As in the cortex, no change with age was observed in Wistars (Figures 3B and 3D).

### T2D has no effect on the density of CR-positive neurons in the neocortex and striatum

**Figure 4:**
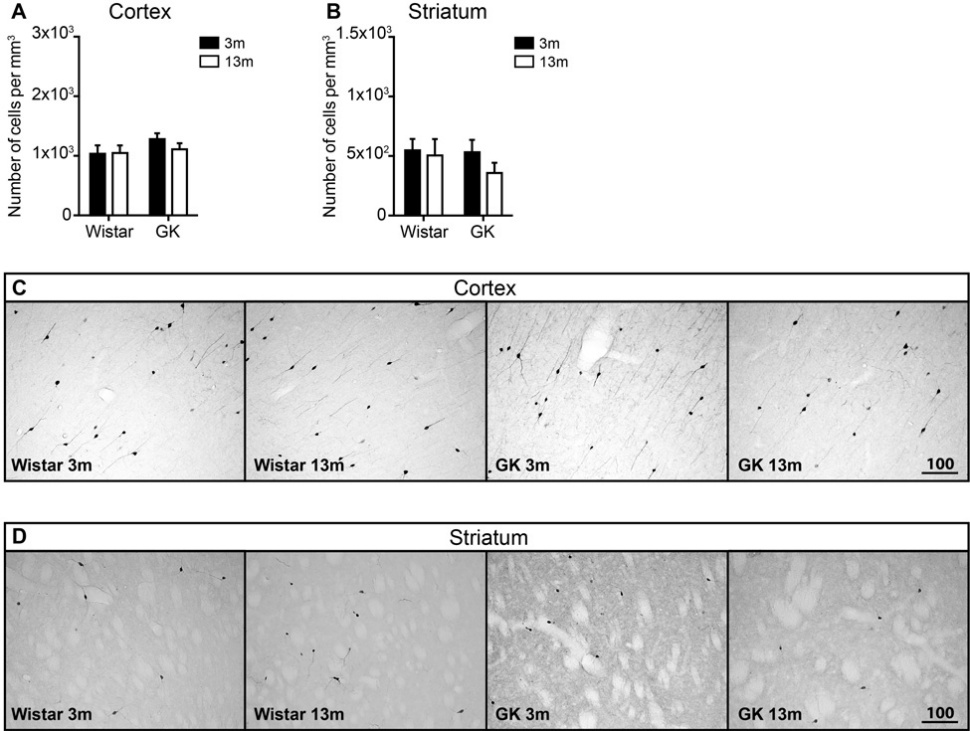
The effect of T2D on the density of CR-positive neurons in the neocortex and the striatum during aging The density of CR-positive neurons in the neocortex (**A**) and the striatum (**B**). The representative images of CR immunoreactivity. Scale bar on panels (**C**) and (**D**) equals 100 μm. Two-way ANOVA was used to determine whether diabetes modified the effect of normal aging observed in Wistar rats. Two-way ANOVA was followed by Tukey's multiple comparison test to determine the differences between experimental groups; 3 months old (3m), 13 months old (13m).

No significant change in the density of CR-positive interneurons with aging or diabetes was observed in either T2D GK or Wistar rats (Figures 4A and 3C) following the two-way ANOVA.

In the striatum, there was a noticeable trend towards the reduction in CR-positive cell density in 13-month-old GK rats compared with 3-month-old GK rats (a 32% reduction), which failed to reach significance after correction for multiple comparisons (Figures 4B and 4D). There was no change in the density of CR-positive cells in Wistar rats (Figures 4B and 4D).

### T2D has no effect on the density of PV-positive neurons in the neocortex and striatum

**Figure 5:**
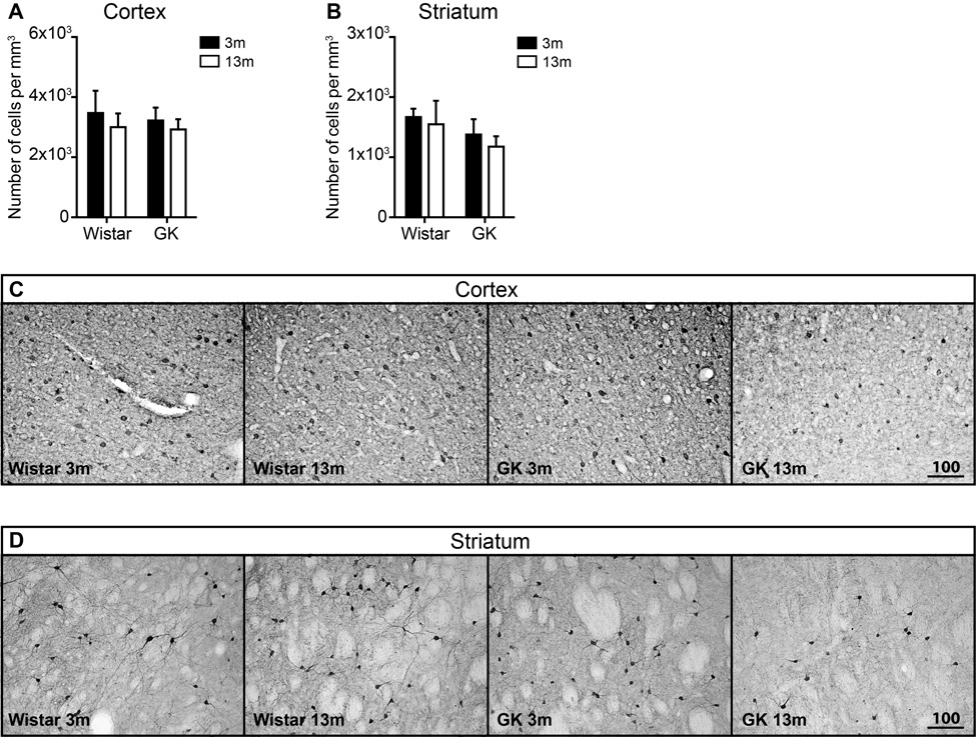
The effect of T2D on the density of PV-positive neurons in the neocortex and the striatum during aging The density of PV-positive neurons in the neocortex (**A**) and the striatum (**B**). The representative images of PV immunoreactivity. Scale bar on panels (**C**) and (**D**) equals 100 μm. Two-way ANOVA was used to determine whether diabetes modified the effect of normal aging observed in Wistar rats. Two-way ANOVA was followed by Tukey's multiple comparison test to determine the differences between experimental groups; 3 months old (3m), 13 months old (13m).

We recorded no significant difference in the density of PV-positive neurons in the neocortex of GK compared with Wistar rats in either age group (Figures 5A and 5C).

In the striatum, the results of the two-way ANOVA showed no statistically significant interaction between age and diabetes. However, there was a notable trend (*P*=0.09) towards the reduction in the density of PV-positive neurons in 13-month-old GK rats in comparison with 3-month-old GK rats (16% reduction) although this did not reach statistical significance (Figures 5B and 5D).

### The volume of parvalbumin positive neurons in the striatum was increase in GK rats

**Figure 6:**
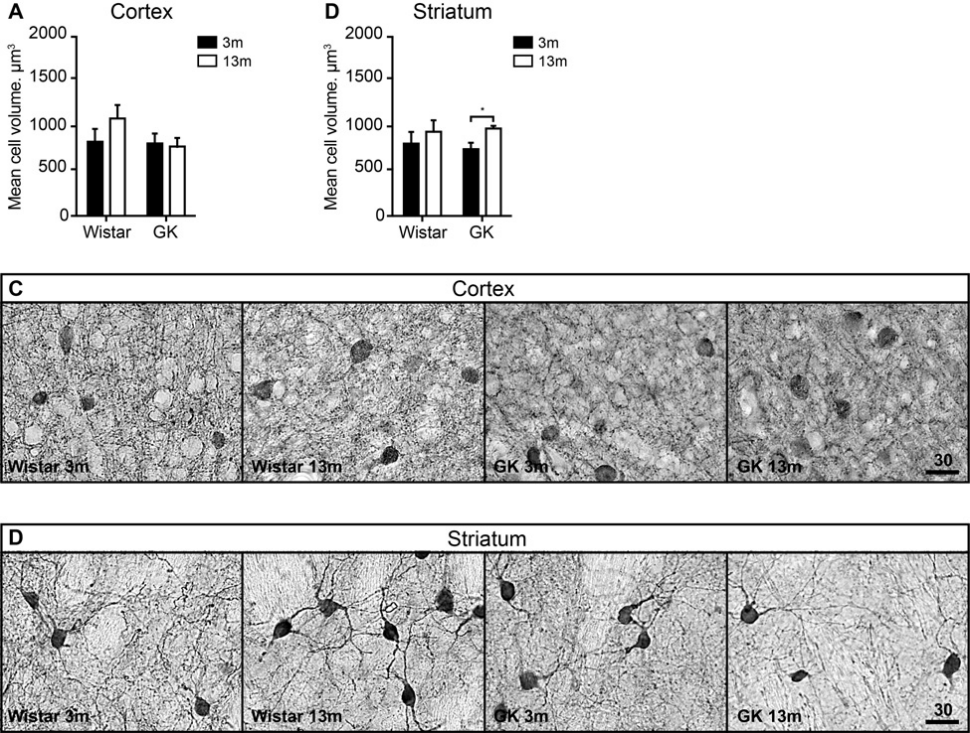
The effect of T2D on the average soma volume of PV-positive neurons in the neocortex and the striatum during aging The average soma volume of PV-positive neurons in the neocortex (**A**) and the striatum (**B**). The representative images of PV immunoreactivity. Scale bar on panels (**C**) and (**D**) equals 30 μm. Two-way ANOVA was used to determine whether diabetes modified the effect of normal aging observed in Wistar rats. Two-way ANOVA was followed by Tukey's multiple comparison test to determine the differences between experimental groups. Error bars indicate means±S.D.; * denotes *P*<0.05; 3 months old (3m), 13 months old (13m).

In the neocortex, no significant change in the volume of PV-positive interneurons with aging or diabetes was observed in either T2D GK or Wistar rats (Figures 6A and 6C) following the two-way ANOVA. However, the average PV-positive cell volume in the 13-month-old Wistar showed a strong trend towards an increase in comparison with 3-month-old Wistars (Figures 6A and 6C).

A similar trend was observed in the striatum of Wistar rats (Figure 6B). In T2D GK rats, the average PV-positive cell volume increase in the striatum was much more pronounced (approximately 30% increase) and statistically significant (*P*<0.01) (Figure 6B). Because both strains showed similar patterns of PV-positive cell volume growth during aging, no statistically significant interaction between age and diabetes was detected by two-way ANOVA, despite the fact that in GK the difference was significant.

### Ex-4 partially counteracted the effect of T2D in the striatum

The treatment with Ex-4 had no effect on the number of GAD67, CR and PV-positive cells in either striatum or neocortex (results not shown).

**Figure 7:**
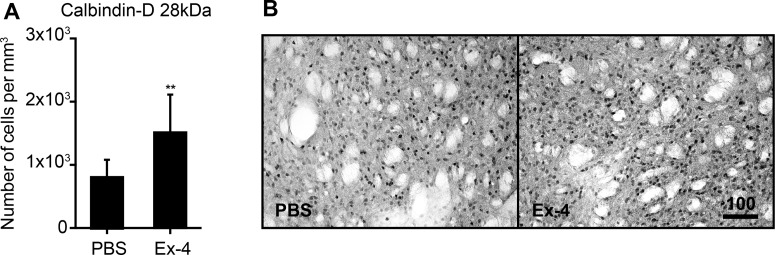
Effect of GLP-1R activation of on the density of CB-positive neurons in the striatum of middle-aged GK rat The number of CB-positive cell in middle-aged T2D GK rats after 6 weeks of PBS or Ex-4 treatment (**A**). The representative images of CB immunoreactivity (**B**). Scale bar on panel (B) equals 100 μm. Student's *t* test was used. The differences were considered significant at *P*<0.05. Error bars indicate means±S.D.; ** denote *P*<0.01.

Ex-4 dramatically increased the density of CB-positive cells in the striatum (90% increase) of GK rats (*P*<0.01) (Figures 7A and 7B). In neocortex, no effect of Ex-4 treatment on CB-positive neurons was recorded (results not shown).

Although increased inflammation in GK rats has been shown [41,42], potential anti-inflammatory effect of Ex-4 in these rats has not been previously investigated. To determine whether the positive effect of Ex-4 on CB-positive cells in the striatum could be related to decreased inflammation in Ex-4 treated rats, we analysed serum cytokines levels. However, we could not detect significant differences between GK rats treated with Ex-4 and vehicle (Table 1).

**Table 1:** Serum cytokine levels after Ex-4 treatment for 6 weeks Samples, below detection limit, were assigned a value corresponding to half of the sensitivity of the assay. Numbers of samples below detection limit IL-1β, 4/19; MCP-1, 0/19; IL-6, 15/19; IL-10, 0/19 and TNFα, 8/19. Data expressed as mean±S.D., pg/ml.

Group	IL-1β	MCP-1	IL-6	IL-10	TNFα
PBS (*n*=11)	6.4±5.0	2058±424	4.6±1.2	58±30	11.4±11.2
Ex-4 (*n*=8)	6.6±5.9	1987±753	5.2±1.0	63±31	16.0±23.4

## DISCUSSION

The aim of the present study was to determine the effect of T2D on GABAergic neurons in the neocortex and the striatum during aging and whether GLP-1R activation could prevent/reverse the identified T2D effect. We show that T2D reduced the density of GAD67-positive neurons in the striatum and of CB-positive neurons in both neocortex and the striatum. In addition, PV-positive neuron volume was significantly increased in GK rats, although it could not be statistically determined whether this effect was related to T2D. Finally, chronic GLP-1R activation by Ex-4 recovered the decrease in CB-positive neuronal density in the striatum.

The pathophysiological mechanisms behind the harmful effects of T2D in the brain are yet to be identified. The characteristic hallmarks of T2D such as insulin resistance, hyperglycaemia, oxidative stress and inflammation are likely involved [13,43–46]. Additionally, several studies have shown that T2D can induce cerebral microvascular disease, which may also lead to neuronal damage [14,47]. All these factors may be linked to brain damage, impaired cognitive function and increased neurodegenerative processes in T2D. However, most of the research in the field has focused on AD and hippocampus. Thus, which specific brain structures and neuronal cell types are affected by T2D needs to be further studied. The strong association of T2D with early cognitive decline and sensorimotor problems led us to hypothesize that T2D can induce pathophysiological changes in specific neural cells and brain areas responsible for these functions. In order to test this hypothesis, we quantitatively evaluated the neuronal composition of the neocortex and the striatum with focus on GABAergic neurons, which play an essential role in the inhibitory modulation of the neuronal activity of these areas [25–27,48,49].

The potential link between metabolism and GABA signalling was already suggested in the 80s by Palovcik et al. [50] and more recent research has shown that metabolic hormones modulate the GABA signalling in different types of neurons [35].

We found significantly lower density of GAD67-positive neurons in the neocortex of GK rats compared with Wistars at both 3- and 13-months. Aging did not modulate the density of GAD67-positive neurons in the neocortex of either rat strain (Figure 2A). Although the earlier involvement of T2D in reducing the density of GAD67-positive neurons in GK rats cannot be fully dismissed, it is unlikely, considering the fact that the density did not changed further at 13 months. It is more plausible that the differences between GK and Wistar rats are determined by the strain and not by the T2D.

In the striatum, we recorded a statistically significant age-dependent increase in the density of GAD67-positive cells in Wistars and decrease in GK rats with aging (Figure 2B). Increase in GAD67 immunoreactivity in the non-diabetic striatum indicates increased GABA production that has been previously observed in aged rats and suggested to be a characteristic feature of normal aging [51]. Thus, the reduced GAD67 immunoreactivity in the striatum of middle-aged T2D GK rats could point towards an abnormal decrease in striatal GABA levels and impairment of inhibitory modulation of striatal neuronal activity in these animals, which in turn could be speculatively linked to early sensorimotor complications in T2D or delayed recovery after stroke [52].

A significant portion of GABAergic interneurons is characterized by the expression of the CaBPs CB, CR and PV. These proteins are involved in calcium buffering and transport, are closely regulated along aging and disease and may play crucial role in maintaining the health of the CNS [53].

The density of CB-positive neurons in the neocortex and striatum was significantly reduced in the middle-aged GK rats compared with young GK or Wistars of both age groups (Figures 3A and 3B). Interestingly, we have recently reported decreased number of CB-positive neurons in the piriform cortex of middle-aged GK rats [24]. Besides reduced cell density (approximately 50% compared with young), a clear reduction (visual observation without quantification) in neurite branching was also evident in the neocortex of middle-aged T2D GK rats (Figures 3A–3C). CB expression in neocortex is localized in inhibitory interneurons, namely in “bursting interneurons” (also expressing PV) [49,54], which play an important role in temporal coordination of pyramidal cell output [54]. Reduced CB expression and neurite branching in middle-aged T2D GK rat neocortex could indicate impaired modulation of cortical excitatory circuits, thus leading to early cognitive decline and sensorimotor complications in T2D.

Oppositely to the neocortex, the majority of striatal CB-positive neurons are not interneurons but medium-size spiny neurons [55]. These neurons are mostly localized in the matrix compartment and project to the *substantia nigra pars reticulata* [56], thus being involved in the activity modulation of dopaminergic neurons. Such reduction in CB-positive neuronal density in the striatum of GK rats (Figures 3B–3D) could be indicative of disrupted calcium homoeostasis in the T2D striatum, that in turn could impair the normal functioning of these cells and potentially affect normal neurological functioning. Indeed, reduced CB mRNA has been reported in several brain structures along aging as well as in a variety of neurodegenerative conditions [57–60]. We can speculate that reduction in CB expression in the striatal matrix compartment projection neurons is indicative of pathological changes that could lead to early impairment of the basal ganglia motor loop in T2D, thus potentially explaining faster development and more severe motor dysfunction observed in PD patients with prior T2D [20].

We did not record statistically significant T2D-related changes during aging in the density of CR- or PV-positive cells in either neocortex or the striatum (Figures 4A and 5B, and Figures 5A and 5B respectively). However, a trend towards the reduction in both of these interneuron subtypes was evident in middle-aged GK rats, especially in the striatum (Figures 4B and 5B). These results suggest that CR and PV interneurons could be more resistant than CB-positive neurons to the effects induced by T2D and that only a longer exposure to the diabetic disease could also affect these neuronal populations. One limitation of the present study is that we did not have older groups of rats (for instance 24 months old) where additional effects induced by T2D could have been detected.

We measured the average cell volume of PV-positive neurons in the neocortex and the striatum. The cell size of neurons has been suggested to positively correlate with the neuronal connections and the target area size [61,62]. The average size of PV-positive neurons in the neocortex of middle-aged Wistar rats was increased by approximately 20% (not statistically significant trend) in comparison with young Wistars, an effect that was not observed in T2D GK rats (Figure 6A). PV-positive interneurons are fast-spiking neurons that demand high energy for normal functioning and play an important role in cortical information processing [48]. A trend towards increased soma volume in normal aging could indicate more network connections and/or increased activity, which are likely inhibited under T2D.

In the striatum, a similar trend towards the increase in the average PV interneuron volume was observed in Wistars with aging. However, in GK rats this increase was significant (Figure 6B). PV-positive interneurons are involved in the activity synchronization of striatal projection neurons [63]. Considering the decrease in GABA (GAD67) (Figure 2B) in the striatum of middle-aged GK rats, which could indicate the reduction in overall inhibitory activity in that structure, we could speculate that the increase in PV-interneuron size could indicate an increased connectivity or inhibitory activity of PV-interneurons as a compensatory mechanism to balance the likely overall decrease in GABAergic inhibitory signalling under T2D. Because similar patterns of PV-interneuron volume growth were also observed in the striatum of non-diabetic Wistars, a statistically significant interaction between age and diabetes was not detected by two-way ANOVA. However, it is plausible that PV-interneuron volume growth is a part of normal aging process, which is further amplified by the T2D.

In order to clarify whether the observed reduction in GAD67- (Figure 2B) and CB-positive neuronal density (Figures 3A and 3B) was caused by cell loss or reduction in protein expression, we quantified the general density of neurons in the neocortex and the striatum by using Nissl staining and stereology methods. We did not detect any changes in the total neuronal density either with age or with T2D (Figures 1B and 1C), indicating that no significant neuronal loss has been induced by T2D. At the first glance, this observation seems to contradict our previous work, where we saw 5–7% decrease in the total number of NeuN-positive neurons in the neocortex of middle-aged, T2D GK rats. This decrease in NeuN-positive neurons was accompanied by reduced or abnormal NeuN expression in the neurons of the neocortex [39]. NeuN is a product of *Fox-3* gene and has been suggested to play a role in neural cell differentiation and development [64], although its functions in mature neurons is unknown. In a recent work, we have reported the reversal of abnormal NeuN expression in the neurons of the piriform cortex by pharmacological GLP-1R activation [24]. Considering these recently published data and the fact that we did not detect changes in neuronal density based on Nissl staining in that study, it is likely that T2D does not induce neuronal loss as measured by NeuN counting, but may rather reduce NeuN expression leading to reduced neuronal counts based on this marker. The absence of significant neuronal loss of the neocortex and the striatum in T2D is further suggested by no detectable differences in the number of TUNEL and cleaved caspase-3 positive cells between GK and Wistar rats in both young and middle-aged rats (results not shown).

We have recently shown the positive effect of GLP-1R activation on the number of CB-positive neurons in the piriform cortex of middle-aged T2D GK rats [24]. Similarly, the GLP-1R activation by Ex-4 increased the density of CB-positive neurons in the striatum, but not in the neocortex of GK rats (Figure 7). As indicated above, CB-positive cells are typically GABAergic interneurons in the neocortex whereas mostly medium-sized projection neurons in the striatum. Thus, our data indicate that GLP-1R activation specifically counteracts the effects of T2D on CB-expression in the striatal projection neurons but not in neocortical interneurons. Striatum plays an important role in motor control and is affected in neurodegenerative diseases such as PD, Huntington's disease (HD) and stroke. Several studies have shown the beneficial effects of GLP-1R activation on motor function in animal models of PD [65–67], HD [68] and improved outcome after stroke [69]. In addition, a previous study showed clinical improvements in PD patients treated with Ex-4 [70]. Thus, the increase in CB expression in the striatum after GLP-1R activation could represent one of the contributing mechanisms in the neuroprotective efficacy mediated by GLP-1R activation. Indeed, neuroprotection by up-regulated CB has been previously demonstrated in animal models of stroke [71].

Previous studies suggest that peripheral inflammation is increased in GK rats [41,42,72] and that GLP-1R activation can decrease inflammation in humans with T2D [41]. Given this background, we sought to investigate whether the beneficial effect on CB-positive neurons by GLP-1R activation could be due to decreased inflammation. We did not find any evidence for Ex-4 to influence cytokine levels. However, it should be kept in mind that in the present study we analysed serum samples and this might not reflect the cytokine levels locally in the brain.

In conclusion, our results show that T2D specifically affects the neocortex and the striatum on different neuronal populations that include GABAergic interneurons and CB-positive neurons. It is likely that these T2D-induced changes may have negative influence on the normal functioning of the GABAergic inhibitory system in these structures. If so, the identified effects could play a role in the early development of cognitive and sensorimotor impairments in T2D patients, as well as in the decreased recovery following brain injuries such as stroke. The efficacy data showing that GLP-1R activation can strongly counteract the T2D-induced CB down-regulation in the striatum provides new knowledge about the specific cellular targets of this class of T2D drugs in the CNS. Whether this finding could have therapeutic implications for the treatment of CNS complications in T2D, where striatal function is involved, remains to be investigated.

## Abbreviations

AD: Alzheimer's diseases
CaBP: calcium-binding protein
CB: calbindin
CNS: central nervous system
CR: calretinin
Ex-4: exendin-4
GABA: γ-aminobutyric acid
GAD67: glutamic acid decarboxylase-67
GK: Goto-Kakizaki
GLP-1R: glucagon-like peptide-1 receptor
HD: Huntington's disease
IHC: immunohistochemistry
IL-1β: interleukin 1 beta
IL-6: interleukin 6
IL-10: interleukin 10
MCP-1: monocyte chemoattractant protein-1
PD: Parkinson's disease
PFA: paraformaldehyde
PV: parvalbumin
T2D: Type 2 diabetes
TNFa: tumor necrosis factor alpha
